# Coronary Artery Calcium Score–Weighted Clinical Likelihood Model Performance in Patients with Stable Chest Pain and Coronary Artery Calcium Scores of Zero

**DOI:** 10.31083/j.rcm2503085

**Published:** 2024-03-04

**Authors:** Yahang Tan, Chang Liu, Tao Chen, Yina Li, Chengjian Wang, Jia Zhao, Jia Zhou

**Affiliations:** ^1^Department of Cardiology, Beijing Chaoyang Hospital, Capital Medical University, 100069 Beijing, China; ^2^Heart Center and Beijing Key Laboratory of Hypertension, Beijing Chaoyang Hospital, Capital Medical University, 100069 Beijing, China; ^3^Clinical School of Thoracic, Tianjin Medical University, 300203 Tianjin, China; ^4^Department of Cardiology, Tianjin Chest Hospital, 300222 Tianjin, China; ^5^Department of Emergency, Hebei Petrochina Central Hospital, 065000 Langfang, Hebei, China

**Keywords:** risk assessment strategy, stable chest pain, coronary artery calcium score, coronary computed tomography angiography, coronary artery calcium score-weighted clinical likelihood model

## Abstract

**Background:**

For individuals with persistent stable chest pain (SCP) and a coronary artery calcium score (CACS) of 0, it might be challenging to establish the best risk assessment method for determining the individuals who will not benefit from further cardiovascular imaging testing (CIT). Thus, we investigated the CACS-weighted clinical likelihood (CACS-CL) model in SCP patients with a CACS of 0.

**Methods:**

Thus, to assess SCP, we originally enrolled 14,232 individuals for CACS and coronary computed tomography angiography (CCTA) scans between January 2016 and January 2018. Finally, patients with a CACS of 0 were included and followed up ​until January 2022. According to the established CACS-CL cutoffs of 15% and 5%, the associations between coronary artery disease (CAD) and major adverse cardiovascular events (MACEs) in risk groups were evaluated, alongside the net reclassification improvement (NRI).

**Results:**

Of the 6689 patients with a CACS of 0, the prevalence of CAD increased significantly (*p* < 0.0001) in patients with higher CACS-CL. However, there was no significant difference in the CAD distribution (*p* = 0.0637) between patients with CACS-CL <5% and 5–15%. The association between the CACS-CL = 15%-determined risk groups and the occurrence of MACEs was stronger than for a CACS-CL = 5% (adjusted hazard ratio (HR): 7.24 (95% CI: 1.93–16.42) versus 3.68 (95% CI: 1.50–8.26)). Compared with the cutoff for CACS-CL = 5%, the NRI was 10.61% when using a cutoff for CACS-CL = 15%.

**Conclusions:**

Among patients with an SCP and CACS of 0, the CACS-CL model provided accurate predictions of CAD and MACEs. Compared to the cutoff for CACS-CL = 5%, the cutoff for CACS-CL = 15% seemed to be more effective and safer for deferring further CIT.

**Clinical Trial registration:**

NCT04691037.

## 1. Introduction

Stable chest pain (SCP) in patients is suggestive of chronic coronary syndrome (CCS). Cardiac imaging testing (CIT) is widely employed to assess the presence of obstructive coronary artery disease (CAD) [1, 2]. Although coronary computed tomography angiography (CCTA) is increasingly recognized as the first-line CIT, per recently published guidelines [1, 2], an increasing body of clinical trials has demonstrated that most patients referred for CCTA as well as other cardiac imaging tests presented no adverse clinical events when the results were negative [3, 4, 5, 6]. As the desire to get the most out of limited resources increases, a lively discussion has appeared regarding how to enhance risk assessment to better choose patients for whom further CIT should be postponed [7, 8, 9, 10].

In this setting, there is a resurgence in interest in using the coronary artery calcium score (CACS) as part of the initial clinical decision-making process, to provide a more accurate evaluation for subsequent CIT [7, 11, 12, 13]. A recent meta-analysis of patients with SCP showed that a CACS of 0 had a negative predictive value of 97% for ruling out obstructive CAD [11]. However, substantial research has consistently established that a significant interaction exists between CACS and risk variables in predicting obstructive CAD and major adverse cardiovascular events (MACEs), while atherosclerosis could not be safely excluded in those with a high-risk factor burden but a CACS of 0 [14, 15, 16, 17]. Thus, considerable work is still needed to improve the CACS-based paradigm regarding risk assessment for SCP, particularly in patients with a CACS of 0.

Using a large cohort consisting of 41,177 symptomatic patients who underwent CCTA from 2008 to 2017, Winther *et al*. [18] developed a CACS-based tool to estimate the clinical likelihood (CL) of obstructive CAD, which included CACS over and above age, sex, symptoms, and other traditional cardiac risk factors. The diagnostic and prognostic values for this CACS-CL model have been validated in several external cohorts [8, 18, 19] and have been recommended by recent guidelines [1, 2]. However, to date, limited data exist on the performance of the CACS-CL model among patients with a CACS of 0. Consequently, the present study aimed to investigate the diagnostic and prognostic values for the CACS-CL model, as well as the effectiveness of current risk assessment strategies based on different cutoffs of CACS-CL, to optimize downstream referrals for CIT in a CCTA-based cohort comprising patients with SCP and a CACS of 0.

## 2. Materials and Methods

### 2.1 Study Cohort

**Fig. 1.**
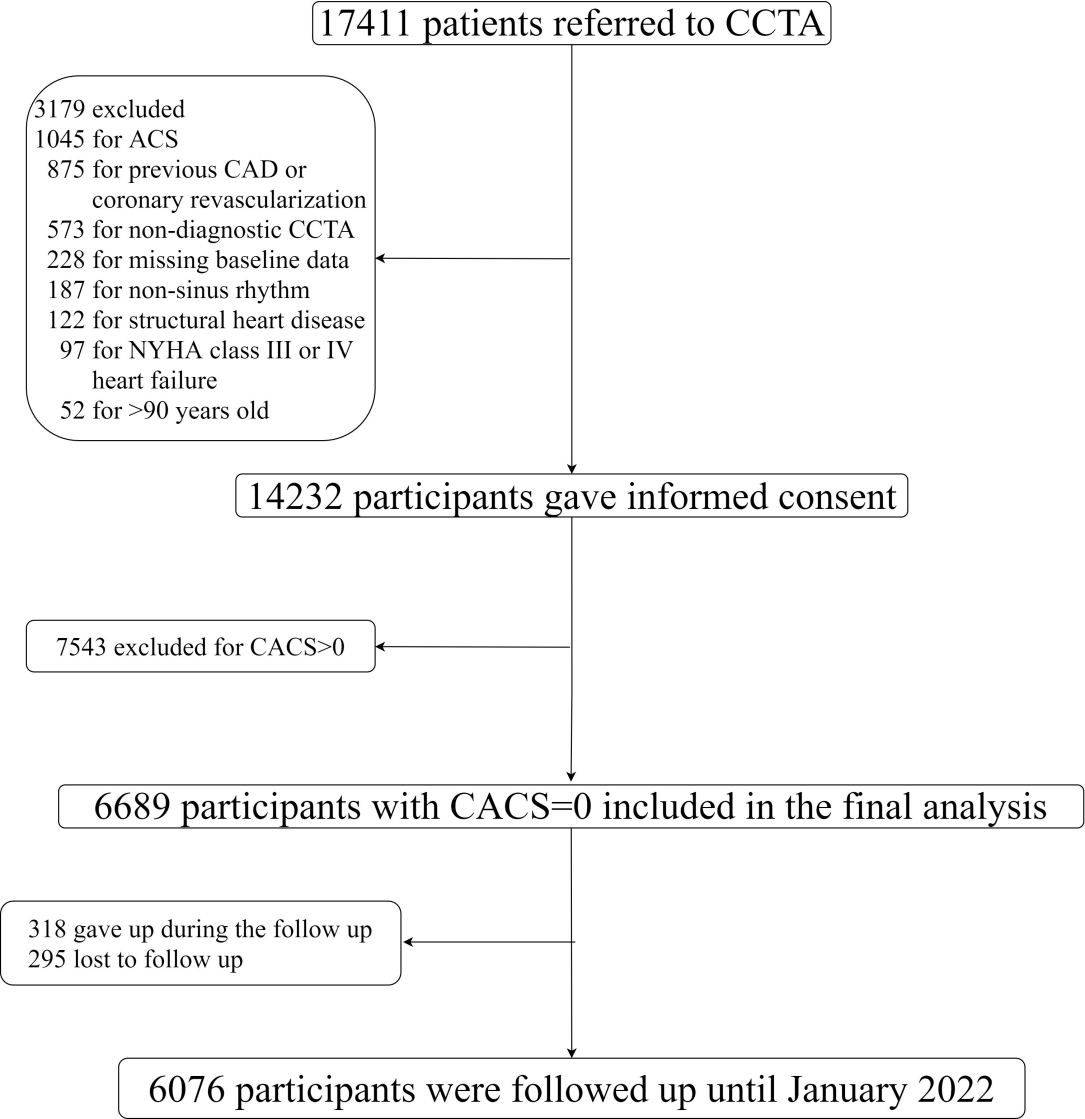
**Flow diagram**. CCTA, coronary computed tomography angiography; ACS, acute coronary syndrome; CAD, coronary artery disease; CACS, coronary artery calcium score; NYHA, New York Heart Association.

Briefly, the CCTA Improves Clinical Management of Stable Chest Pain (CICM-SCP) registry is an ongoing cohort of patients who were referred to CCTA as the first-line CIT to assess their SCP in relation to CCS (ClinicalTrials.gov Identifier: NCT04691037). As shown in Fig. 1, during a period of 24 months, between January 2016 and January 2018, 14,232 patients were finally enrolled in this registry, while details on the registry have been previously described [8, 17]. In the present analysis, patients with a CACS of 0 were included and followed up until January 2022. This study was approved by the Ethics Committees at local institutions and all participants provided informed consent.

### 2.2 Procedure and Postprocessing Analysis of CACS and CCTA

The scanning, as well as interpretation of CACS and CCTA, were conducted as described previously [8, 17]. Patients with a CACS of 0 were included in the present study. Based on the Coronary Artery Disease–Reporting and Data System [20], the maximal degree of coronary diameter stenosis was defined as no CAD (0%), nonobstructive CAD (1–49%), and obstructive CAD (≥50%).

### 2.3 Baseline Clinical Data

Baseline clinical data, including age, sex, diabetes, hypertension, hyperlipidemia, smoking, family history of CAD, and symptoms were collected and defined as described previously [8, 17]. Hypertension was defined as blood pressure ≥140/90 mmHg or the use of anti-hypertension medication. Hyperlipidemia was defined as total cholesterol levels of ≥220 mg/dL, low-density lipoprotein cholesterol of ≥140 mg/dL, fasting triglycerides of ≥150 mm/dL, or receiving treatment with oral lipid-lowering agents. Diabetes was defined as fasting glucose levels over 7 mmol/L or treatment currently with either diet, oral glucose-lowering agents, or insulin. Smoking was defined as currently smoking or having smoking in the past 6 months. A family history of CAD was defined as a diagnosis of the disease in a male first-degree relative before 55 years of age or in a female first-degree relative before 65 years of age. Typical angina was defined as having 3 characteristics [1]: (1) substernal discomfort of characteristic quality, (2) precipitated by physical exertion or emotion, and (3) relieved with rest or nitroglycerin within 10 min. Atypical angina was defined as having 2 of the 3 defined characteristics. Nonanginal chest pain was characterized as chest pain or discomfort that met 1 or 0 of the 3 defined characteristics.

### 2.4 CACS-CL and Risk Groups

CACS-CL was estimated for each patient using the variables mentioned above (age, sex, diabetes, hypertension, hyperlipidemia, smoking, family history of CAD, and symptoms) as well as CACS based on plugins to the statistical software, packaged from the original study by Winther *et al*. [18]. The CADPTP models for implementation in R were available at https://github.com/CardioLab/cadptp/tree/master/R. Based on the recommendations of recent guidelines, CIT is not recommended in low-risk patients and should be referred only to high-risk patients [1, 2]. Thus, we selected two different cutoffs for CACS-CL (5% and 15%) to classify patients into either the low (CACS-CL <5% and ≤15%) and high (CACS-CL ≥5% and >15%) risk group, respectively.

### 2.5 Follow-Up of MACEs

The research endpoint and the collection of follow-up data were previously defined in detail [8, 17]. Briefly, all patients were followed at the 6th, 12th, 24th, 36th, 48th, 60th, and 72nd months after CCTA. MACEs were defined as a composite of all-cause death and nonfatal myocardial infarction (MI). All-cause death was used rather than cardiovascular death to eliminate the competing events of cardiovascular and noncardiovascular mortality, as well as the need for possibly difficult adjudication of causes of death, especially given the relatively low mortality. All MACEs were adjudicated via a review of the follow-up information and medical records by an independent clinical event committee who were blinded to other study data.

### 2.6 Statistical Methods

All data analyses were performed using MedCalc (version 15.2.2; MedCalc Software, Mariakerke, Belgium) and R (version 3.2.4; R Foundation for Statistical Computing, Vienna, Austria). A *p* value < 0.05 (two-tailed) was considered significant. The ANOVA or Mann–Whitney U test was used to evaluate the differences in continuous variables as appropriate. The χ^2^ test or Fisher’s exact test was used to evaluate the differences in the categorical variables as appropriate. The discrimination and calibration of the CACS-CL model were assessed by the area under receiver operating characteristic curve (AUC) and Hosmer–Lemeshow goodness-of-fit statistic (H-L χ^2^) [21]. Net reclassification improvement (NRI) was assessed in a reclassification table and used to determine how strategies using different CACS-CL cutoffs reclassified patients into various risk groups compared with each other [21]. The cumulative MACE-free survivals were estimated using Kaplan–Meier curves and were compared by the log-rank test. We used Cox proportional hazard regression models to calculate the adjusted hazard ratios (HRs) and 95% confidence intervals (CIs), which assessed the association of risk groups to the onset of the first MACE. The models were adjusted for age, sex, cardiovascular risk factors (diabetes, hypertension, hyperlipidemia, smoking, family history of CAD), symptoms, and CACS.

## 3. Results

### 3.1 Baseline Characteristics of Study Population

Baseline characteristics according to CACS-CL (<5%, 5–15%, and >15%) are shown in Table 1. The CCIM-SCP cohort contained 14,232 symptomatic patients, 6689 (47%) of whom had a CACS of 0 and were included in the current investigation. The average age was 57.26 years, and 57% of the participants were men. Of the 6689 patients with a CACS of 0, 81% were classified as CACS-CL <5%, 15% as CACS-CL 5–15%, and 4% as CACS-CL >15%. All baseline characteristics were significantly different across the three CACS-CL categories (*p* < 0.0001 for all comparisons).

**Table 1.** **Baseline characteristics by CACS-CL**.

		All	CACS-CL			*p*
		(n = 6689)	<5% (n = 5419)	5–15% (n = 1023)	>15% (n = 247)	
Age*		57.26 ± 12.76	56.38 ± 12.94	59.43 ± 13.58	67.59 ± 17.36	<0.0001
Male		3813 (57)	3035 (56)	608 (59)	170 (69)	<0.0001
Diabetes		736 (11)	542 (10)	142 (14)	52 (21)	<0.0001
Hypertension		2408 (36)	1842 (34)	412 (40)	154 (62)	<0.0001
Hyperlipidemia		1940 (29)	1517 (28)	327 (32)	96 (39)	<0.0001
Smoking		1605 (24)	1138 (21)	339 (33)	128 (52)	<0.0001
Family history of CAD		1204 (18)	921 (17)	203 (20)	79 (32)	<0.0001
Symptoms						<0.0001
	Nonanginal anginal	3478 (52)	2980 (55)	451 (44)	47 (19)	
	Atypical anginal	2542 (38)	1897 (35)	477 (47)	168 (68)	
	Typical anginal	669 (10)	542 (10)	95 (9)	32 (13)	

Values are presented as n (%) unless stated otherwise. CACS-CL, coronary artery calcium score-weighted clinical likelihood; CAD, coronary artery disease. * years, mean ± standard deviation.

### 3.2 Validation of CACS-CL Model

**Fig. 2.**
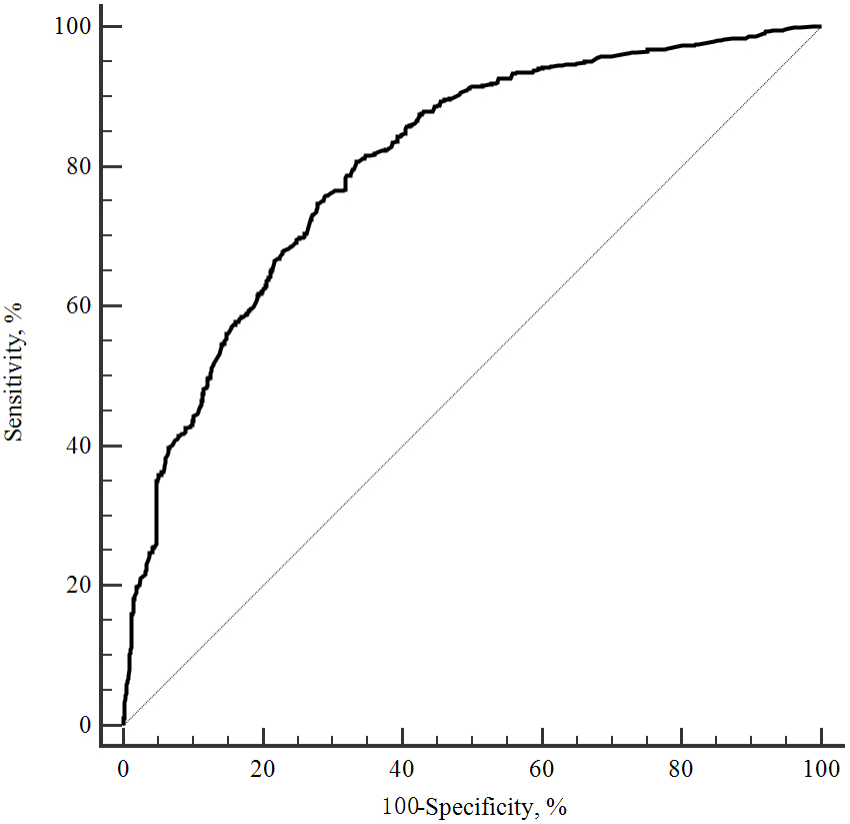
**ROC curve for the CACS-CL model to predict obstructive CAD **. ROC, receiver operating characteristic; CACS-CL, coronary artery calcium score-weighted clinical likelihood; CAD, coronary artery disease.

**Fig. 3.**
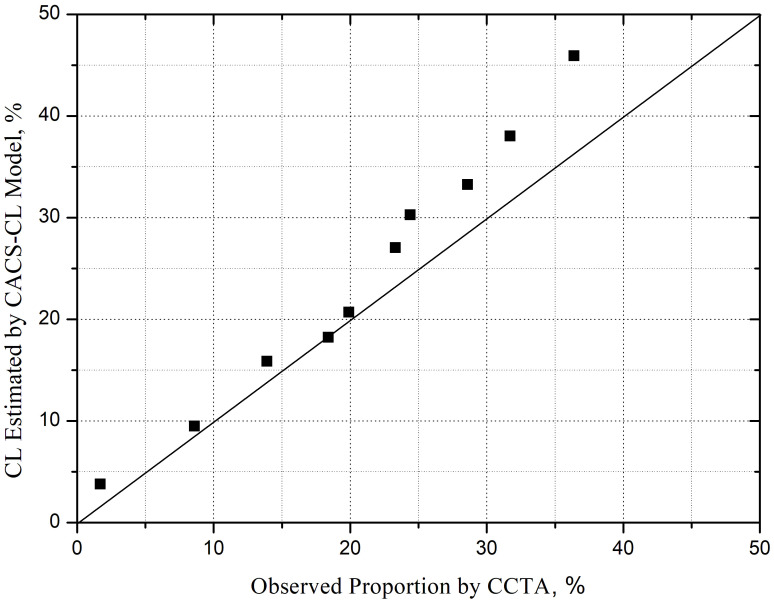
**Comparison of CACS-CL and proportion of obstructive CAD on CCTA by deciles of CACS-CL**. CACS-CL, coronary artery calcium score-weighted clinical likelihood; CAD, coronary artery disease; CCTA, coronary computed tomography angiography; CL, clinical likelihood.

The receiver operating characteristic curve for the CACS-CL model is presented in Fig. 2. The discrimination of the CACS-CL model was excellent, with an AUC of 0.805 (95% CI: 0.790–0.819, *p* < 0.0001). The calibration plot for the CACS-CL model is illustrated in Fig. 3. Graphically, in the group with negative CACS-CL, the probability of the obstructive CAD was overestimated, resulting in a moderate calibration (H-L χ^2^ = 13.19, *p* < 0.0001).

### 3.3 CACS-CL and CAD on CCTA

**Fig. 4.**
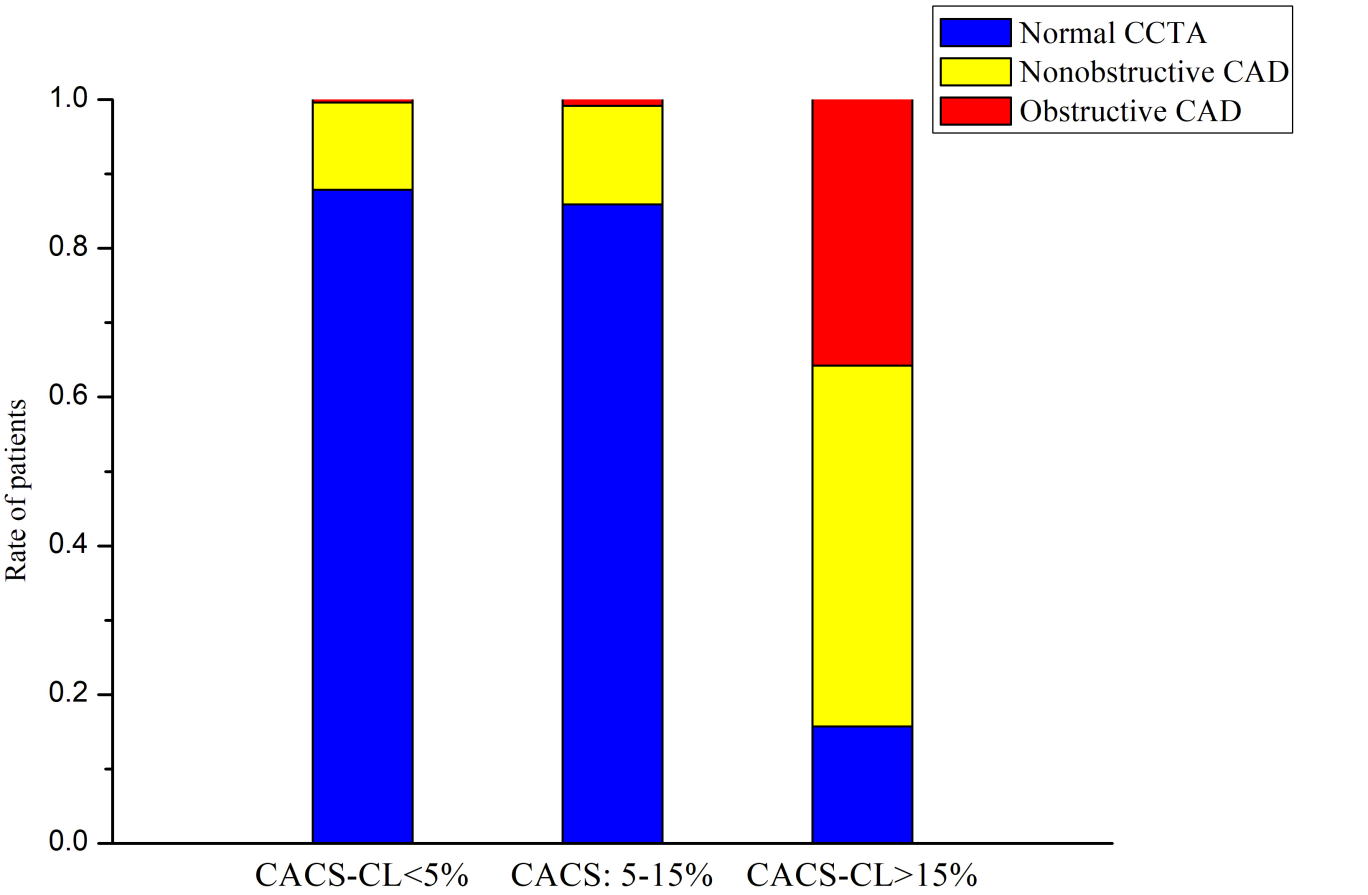
**Distribution of CAD according to CACS-CL (<5%, 5–15%, and >15%)**. CACS-CL, coronary artery calcium score-weighted clinical likelihood; CAD, coronary artery disease; CCTA, coronary computed tomography angiography.

In total, 247 (4%), 1023 (15%), and 5419 (81%) patients had obstructive, nonobstructive, and no CAD, respectively, based on CCTA. As illustrated in Fig. 4, compared to patients with CACS-CL <5% and 5–15%, patients with CACS-CL >15% had more (*p* < 0.0001 for both comparisons) obstructive (36% (219/612) versus 0.4% (21/5204) and 0.8% (7/873)) and nonobstructive CADs (49% (297/612) versus 12% (610/5204) and 13% (116/873)), respectively. However, there was no significant (*p* = 0.0637) difference between patients with CACS-CL <5% and 5–15% in terms of CAD distribution.

### 3.4 Follow-Up of MACEs

During the 5-year follow-up (median: 61 months, interquartile range: 54 to 68 months), 1% (73/6689) of patients experienced MACEs: 19 patients died and 54 patients suffered from nonfatal MI. As shown in Table 2, the rates of the MACEs were 0.6%, 1% (10/863), and 13% in patients with CACS-CL <5%, 5–15%, and >15%, respectively. Among the 32, 95, and 542 typical anginal patients with CACS-CL <5%, 5–15%, and >15%, 23, 5, and 19 experienced MACEs, respectively. Thus, most (64%, 47/73) MACEs occurred in typical anginal patients. Moreover, as shown in Table 2, typical anginal patients with a CACS-CL <15% had similar a rate of MACEs as patients with CACS-CL >15% who have not yet developed typical angina. This suggests that three distinct risk cohorts can be further redefined. Cohort 1 (very low risk) included patients with CACS-CL <15% but without typical angina. The rate of MACEs in this cohort was 0.3% (17/5805). Cohort 2 (moderate risk) included patients with CACS-CL <15% and typical angina or patients with CACS-CL >15% but without typical angina. The rate of MACEs in this cohort was 4.3% (37/852). Cohort 3 (high risk) included patients with CACS-CL >15% and typical angina. The rate of MACEs in this cohort was 59% (19/32).

**Table 2.** **Distribution of MACEs according to CACS-CL and symptoms**.

		CACS-CL		
		<5% (n = 5419)	5–15% (n = 1023)	>15% (n = 247)
MACE		31 (0.6)	10 (1.0)	32 (13)
	Death	11 (0.2)	3 (0.3)	9 (3.6)
	Nonfatal myocardial infarction	20 (0.4)	7 (0.7)	23 (9.4)
Typical anginal		542 (10)	95 (9)	32 (13)
	MACE in these patients	23 (4.2)	5 (5.3)	19 (59)
Atypical + nonanginal anginal		4877 (90)	928 (91)	215 (87)
	MACE in these patients	12 (0.3)	5 (0.5)	9 (4.2)

Values are presented as n (%) unless stated otherwise. CACS-CL, coronary artery calcium score-weighted clinical likelihood; MACE, major adverse cardiovascular event.

**Fig. 5.**
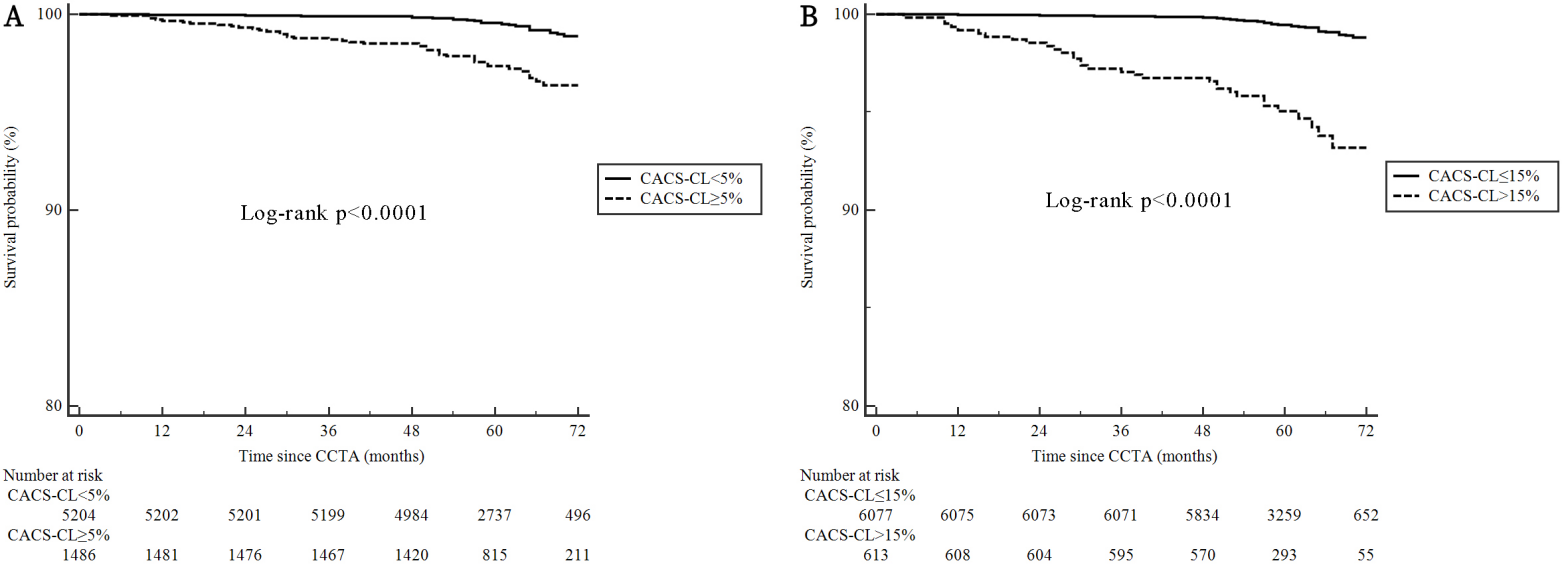
**Cumulative survival probability from MACEs according to different cutoffs for CACS-CL**. (A) CACS-CL ≥5% versus <5%. (B) CACS-CL >15% versus ≤15%. CACS-CL, coronary artery calcium score-weighted clinical likelihood; MACE, major adverse cardiovascular event; CCTA, coronary computed tomography angiography.

Fig. 5 illustrates the Kaplan–Meier estimates for patients surviving free from MACEs. The patients in the high-risk group according to the cutoff for CACS-CL = 5% and 15% had a significantly higher (log-rank *p* < 0.0001 for both) risk of MACEs, respectively, although the association between CACS-CL = 15%-determined risk groups and MACEs was stronger than for CACS-CL = 5% (adjusted HR for CACS-CL >15% versus ≤15%: 7.24, 95% CI 1.93–16.42; adjusted HR for CACS-CL ≥5% versus <5%: 3.68, 95% CI 1.50–8.26).

### 3.5 Reclassification Table and NRI Comparing CACS-CL Cutoffs of 15% and 5% 

Table 3 is the reclassification table for obstructive CAD. Compared to the strategy, which used the cutoff for CACS-CL = 5%, among the 6442 patients without obstructive CAD, 901 patients were correctly reclassified from the high-risk group to the low-risk group. However, 35 patients were incorrectly reclassified from the low-risk group to the high-risk group by the strategy that used the cutoff for CACS-CL = 15%. Among the 247 positive patients, the strategy that used the cutoff for CACS-CL = 15% correctly reclassified one patient from the low-risk group to the high-risk group, although eight patients were incorrectly reclassified from the high-risk group to the low-risk group. Therefore, the NRI of the strategy that used the cutoff for CACS-CL = 15% compared with the strategy that used the cutoff for CACS-CL = 5% was 13.45% for negative patients, –2.84% for positive patients, and 10.61% for all patients. Similarly, when regarding any CAD in Table 4, the NRI was 13.84% for negative patients, –9.68% for positive patients, and 4.16% for all patients.

**Table 3.** **Reclassification table for obstructive CAD comparing CACS-CL cutoffs of 15% and 5%**.

		Risk groups by CACS-CL = 15%		Total	${\mathrm{Reclassification}}^{a}$		${\mathrm{NRI}}^{b}$	*p*
		Low	High		Up	Down		
Risk groups by CACS-CL = 5%								
Negative patients					0.54%	13.99%	10.61%	<0.0001
	Low	5148	35	5183				
	High	901	358	1259				
	Total	6049	393	6442				
Positive ${\mathrm{patients}}^{c}$					0.40%	3.24%		
	Low	20	1	21				
	High	8	218	226				
	Total	28	219	247				

CACS-CL, coronary artery calcium score-weighted clinical likelihood; CAD, coronary artery disease; NRI, net reclassification improvement. ^a^The reclassification of patients by the horizontal strategy was compared to that by the vertical one. ^b^NRI = [P(Up | Positive) – P(Down | Positive)] – [P(Up | Negative) – P(Down | Negative)]. ^c^A positive patient was defined as a patient who had obstructive CAD.

**Table 4.** **Reclassification table for any CAD comparing CACS-CL cutoffs of 15% and 5%**.

		Risk groups by CACS-CL = 15%		Total	${\mathrm{Reclassification}}^{a}$		${\mathrm{NRI}}^{b}$	*p*
		Low	High		Up	Down		
Risk groups by CACS-CL = 5%								
Negative patients					0.20%	14.04%	4.16%	<0.0001
	Low	4562	11	4573				
	High	761	85	846				
	Total	5323	96	5419				
Positive ${\mathrm{patients}}^{c}$					0.32%	10.00%		
	Low	627	4	631				
	High	127	512	639				
	Total	754	516	1270				

CACS-CL, coronary artery calcium score-weighted clinical likelihood; CAD, coronary artery disease; NRI, net reclassification improvement. ^a^ The reclassification of patients by the horizontal strategy was compared to that by the vertical one. ^b^ NRI = [P(Up | Positive) – P(Down | Positive)] – [P(Up | Negative) – P(Down | Negative)]. ^c^ A positive patient was defined as a patient who had any CAD.

## 4. Discussion

The study investigated the performance of the CACS-CL model in a real-world cohort and demonstrated its diagnostic and prognostic efficacies among patients with a CACS of 0, who had been referred to CCTA for SCP suspected of CCS. Compared to the risk assessment strategy with a CACS-CL cutoff of 5%, adopting a 15% cutoff appeared more promising for optimizing CIT referrals in negative CACS patients. Moreover, most MACEs occurred in typical anginal patients, meaning we further developed three distinct risk cohorts based on the distribution of MACEs according to CACS-CL and symptoms, thereby informing doctors of the need to take particular heed when managing these patients, especially if they have a high-risk clinical profile.

A landmark meta-analysis of 79,903 patients with SCP showed that the prevalence of no, nonobstructive, and obstructive CAD among those with a CACS = 0 was 84%, 13%, and 3%, respectively [11], which were consistent with data in the present study. Additionally, a CACS of 0 predicted a low incidence of MACEs [2], which was also determined by this study. These findings all supported the importance of a CACS = 0 in a value-based healthcare delivery model as a “gatekeeper” for further CIT among individuals with persistent SCP. However, several studies found that the diagnostic and prognostic values of a CACS of 0 were also dependent on clinical variables [14, 15, 16, 17]. In line with this, the disease burden of CAD and MACEs was widely distributed across different CACS-CL.

Hence, the investigation of the CACS-CL model, including CACS over and above traditional risk factors, to improve risk assessment in SCP patients with a CACS of 0 was valuable. To the best of our knowledge, this is the first research study to thoroughly verify and compare the CACS-CL model and different CACS-CL cutoffs in SCP patients with a CACS of 0. In this study, despite the possibility of a sub-optimal calibration due to ethnic variance, our findings indicated that the CACS-CL model accurately predicted obstructive CAD and MACEs in SCP patients with a CACS of 0, which was similar to the general SCP patients [8, 18, 19]. Thus, a CACS-CL model-based risk assessment strategy to defer unnecessary CIT may be effective and safe for general SCP patients, even patients with SCP and a CACS of 0 [8, 18, 19].

More fundamentally, among SCP patients with a CACS of 0, which was considered a low-risk population, a better strategy for selecting patients who would benefit from further CIT is required in the contemporary environment of rising healthcare costs [7]. To address this gap, we compared two different CACS-CL cutoffs of 15% and 5% using several measures. The results from the analyses of NRI and Kaplan–Meier estimates all favored a CACS-CL cutoff of 15%, for positive NRI and stronger associations of risk groups with MACEs. Thus, the risk assessment strategy, which used a CACS-CL cutoff of 15% seemed to be more effective and safer at deferring further CIT in SCP patients with a CACS of 0. The superiority in the CACS-CL cutoff of 15% over the 5% cutoff might be attributed to the similar distribution of CAD between patients with CACS-CL <5% and 5–15%.

There were numerous limitations in this study that should be mentioned. Firstly, this study was an observational subset of a large cohort, meaning there were potential selection biases resulting from the clinical decisions of local physicians. Unfortunately, due to the lack of more detailed information from this observational registration, we cannot specifically identify the causes of deaths nor validly differentiate between the Type I and II MIs. Moreover, the change in CACS during the follow-up, which has great clinical significance, could not be collected. Secondly, the analysis focused on whether there was at least 50% coronary diameter stenosis, thereby limiting the identification of individuals benefiting most from revascularization, such as those with left main disease or 3-vessel disease with a maximum degree of coronary diameter stenosis 70% [22]. Thirdly, this study solely targeted negative CACS patients with SCP, meaning these findings should not be generalized to other patient groups. Lastly, larger population sizes and long-term outcome data are essential for validating and confirming the study’s findings.

## 5. Conclusions

Among patients with SCP who are suspected of CCS and possess a CACS of 0, the CACS-CL model accurately predicted CAD and MACEs. Compared to the risk assessment strategy using a cutoff for CACS-CL = 5%, the strategy that used a cutoff for CACS-CL = 15% seemed to be more effective and safer to defer further CIT in SCP patients with a CACS of 0.

## Data Availability

The datasets used and/or analyzed during the current study are available from the corresponding author on reasonable request.
